# Experiences of an internet-based support and coaching model for adolescents and young adults with ADHD and autism spectrum disorder –a qualitative study

**DOI:** 10.1186/s12888-018-1599-9

**Published:** 2018-01-18

**Authors:** Helena Sehlin, Britt Hedman Ahlström, Gerhard Andersson, Elisabet Wentz

**Affiliations:** 1 Gillberg Neuropsychiatry Centre, Institute of Neuroscience and Physiology, University of Gothenburg, Gothenburg, Sweden; 2 Kungsbacka Clinic of Adult Psychiatry, Department of Psychiatry, Kungsbacka, Region Halland Sweden; 3 Department of Health Sciences, University West, Trollhättan, Sweden; 4 Department of Behavioral Sciences and Learning, Linköping University, Linköping, Sweden; 5 Department of Clinical Neuroscience, Karolinska Institute, Stockholm, Sweden

**Keywords:** ADHD, Autism, Coaching, Support, Internet-based, Adolescent, Adult, Qualitative, Experiences

## Abstract

**Background:**

There is a great demand for non-medical treatment and support targeting the needs of adolescents and young adults with autism spectrum disorder (ASD) and attention-deficit/hyperactivity disorder (ADHD). There is also a lack of qualitative studies providing in-depth insight into these individuals’ own experiences within this area. The current study aimed to explore how adolescents and young adults with ADHD, ASD or both experienced taking part in an internet-based support and coaching intervention.

**Methods:**

Sixteen participants with ASD, ADHD or both who had participated in an 8-week internet-based support and coaching model, were interviewed using semi-structured interviews. Data was analyzed using qualitative content analysis.

**Results:**

Analysis yielded three themes; *Deciding to participate*, *Taking part in the coaching process* and *The significance of format*. Various motives for joining were expressed by participants, such as viewing the technology as familiar and appealing and expecting it to be better suited to their situation. There was also a previously unfulfilled need for support among participants. In deciding to take part in the intervention the coaches’ competence and knowledge were considered essential, often in the light of previously negative experiences. Taking part in the coaching process meant feeling reassured by having someone to turn to in view of shared obstacles to seeking and receiving help. The support was used for talking through and receiving advice on matters related to their diagnosis. Findings further revealed appreciation for aspects relating to the format such as communicating through the written word, being in one’s own home and an experience of immediacy. Some disadvantages were voiced including incomplete personal interaction and failing technology. There were also suggestions for greater flexibility.

**Conclusions:**

The in-depth qualitative data obtained from this study suggest that the current model of support and the internet-based format have specific qualities that could play an important role in the support of adolescents and young adults with ADHD and ASD. Although not a replacement for face-to-face interaction, it could be a promising complement or alternative to other support and treatment options.

**Trial registration:**

“Internet-based Support for Young People with ADHD and Autism - a Controlled Study” retrospectively registered in www.clinicaltrials.gov (ClinicalTrials.gov Identifier: NCT02300597) at 2014–11-10.

## Background

Attention-deficit/hyperactivity disorder (ADHD) and autism spectrum disorder (ASD) are both neurodevelopmental disorders with an early childhood debut [1]. Contrary to previous beliefs, recent research has shown that the majority of individuals with ADHD still meet diagnostic criteria in adulthood, affecting 2–4% of the adult population [2–5]. For ASD the prevalence rate for adults is 1% [6]. Although separate disorders ADHD and ASD often share certain cognitive impairments, such as problems with executive function [7]. There is also a considerable overlap between them which has led to a joint conception known as ESSENCE (Early Symptomatic Syndromes Eliciting Neurodevelopmental Clinical Examinations) [1] and to ASD and ADHD no longer being mutually exclusive in the *Diagnostic and Statistical Manual of Mental Disorders* [8].

Awareness about the persistence of symptoms in ADHD and ASD over the life span has increased the demand for treatment and support options specifically targeting the needs of adolescents and adults [9–12]. Medication such as psychostimulants are regarded effective for adult ADHD but are not always sufficient and the number of controlled studies concerning stimulant medication for emerging adults is scarce [13, 14]. Mounting evidence supports the use of multimodal treatment including psychoeducation and cognitive behavioural therapy [15]. Several qualitative studies have shown that despite medication being helpful, adolescents and adults with ADHD in European countries and in the U.S. still report difficulties within several areas, and experience a strong need for psychological treatment or psychoeducation that is often unmet by healthcare [16–18]. Importantly, there are no pharmacological treatments available for core symptoms in ASD, and research into support for the adult population is considerably lacking [9]. Current recommendations include interventions such as cognitive behavioural therapy and social skills training [19]. A qualitative study [10] examining experiences reported by adults with ASD in the U.S. identified factors on several levels (patient/provider/system level) that negatively impacted the accessibility and experiences of received health care for this group.

More recently, interest has increased in coaching to aid individuals with ADHD [20–22]. Coaching is a diverse field and a single operationalized definition is hard to find [22, 23]. Target areas can include individualized support, encouragement and structure, psychoeducation; providing information about ADHD and the outcomes of living with it, as well as developing strategies to handle problems in everyday living [22–25]. A limited number of studies, including a few qualitative ones, have specifically focused on coaching for adolescents and adults with ADHD [24–27]. These previous studies are all important but also acknowledge methodological limitations such as narrow sample sizes and possible confounding variables. However, they show some initial support for the concept of coaching as helpful for individuals with ADHD. A qualitative study concerning the experiences of college students with ADHD also showed a preference for the term “coaching” and strategy-based interventions over ones including the term “therapy” [18]. A few studies have suggested that coaching could be helpful for individuals with ASD within social skills training or as support within an academic setting [28, 29].

Telehealth and internet-based mental health interventions have received increased interest over the past decade as a complement to current treatment options [30–32]. For people with ASD and ADHD core aspects of the condition can sometimes pose a barrier in traditional health care. Problems with eye contact, reading body language and in processing simultaneous perceptual modalities often make face-to-face contact challenging for individuals with ASD [33, 34]. Furthermore, difficulties with structure, motivation, procrastination and time management can lead to high dropout rates and problems with treatment adherence for individuals with ADHD [35, 36]. In remote geographical areas there is often a shortage of practitioners specialized in ASD and/or ADHD. There is almost no published research on internet interventions within mental health for individuals with these disorders. One recent study, however, showed encouraging results for an internet-based cognitive behavioral therapy program designed for adults with ADHD [37].

The critical period of transition from adolescence into adulthood can be difficult for those with ASD and ADHD. Less external structure from home and/or school and increased demands to independently manage the tasks of daily living pose significant executive function challenges for these individuals [18, 24, 38–41]. Therefore, adolescents and young adults with these conditions often require alternatives to medical help to deal with different aspects of their daily life. There is however a great shortage of both qualitative and quantitative studies within this area. Most notably there is a need for qualitative studies examining these individuals’ own perspectives on treatment and support [9, 10, 16, 19].

Finding forms of support that are acceptable and accessible for these individuals is imperative. Towards this objective, the aim of this study was to use a qualitative methodology to examine the experiences of adolescents and young adults with ADHD, ASD or both who had participated in an 8-week internet-based support and coaching intervention.

## Methods

### Design

This qualitative interview study was part of a multicentre study evaluating the intervention “NP Young Coaching”, an internet-based support and coaching model (IBSC) for adolescents and young adults with ASD and ADHD [12]. The intervention (IBSC) took place at two study centres in the southwest of Sweden between the autumn of 2010 and winter 2014. Interviews were carried out up until the spring of 2015.

#### Internet-based support and coaching

The internet-based support and coaching model used was developed by E.W. and tested in a pilot study [12]. The model builds on coaching and psychoeducative techniques in order to facilitate the individual’s understanding and handling of his/her everyday life problems [23, 42]. The model entails using professionals with extensive knowledge about ADHD and ASD. It includes an initial face-to-face meeting with an appointed coach followed by eight weeks of internet-based coaching. Coaching consists of two weekly scheduled chat-sessions á 30–60 min which typically take place from the participants´ homes by using a specially designed chat program. Two sessions (week 3 and week 6 of the intervention) are live meetings with the coach at their home clinic. Coaches and participants can also communicate via an e-mail function. Before the intervention starts, participants choose two preliminary topics that they want to focus on during chat-sessions [12].

### Settings and procedure

#### The IBSC multicentre study

After the pilot study, the model was tested in a multicentre study including participants from a psychiatric outpatient clinic specialized in adults with ASD and ADHD, and a Habilitation service centre serving children, adolescents and adults. Participants were adolescents or young adults, aged 15 to 32 years, with a confirmed DSM–IV diagnosis of ADHD, ASD or both. Participants included both individuals who recently received a neuropsychiatric evaluation as well as individuals who had previously been assigned the diagnosis. All neuropsychiatric evaluations had been performed at well-established clinics with multi-professional teams. Hence, the participants´ symptoms were regarded as leading to clinically significant impairment as described in the diagnostic criteria of the DSM-IV for these disorders. Exclusion criteria were other current serious or dominant disorders (e. g. psychosis, alcohol or substance abuse and/or conduct disorder/antisocial personality disorder). This was established by using the Structured Clinical Interview for DSM-IV Axis I Disorders (SCID-I) [43] and the antisocial personality disorder part of the Structured Clinical Interview for DSM-IV Axis II Disorders (SCID-II) [44]. Major depression was an exclusion criterion if the participant was judged in need of another treatment or not suitable/able to comply with the format of the intervention. Finally, individuals with severe dyslexia and mental retardation/intellectual disability were excluded. A total of 30 individuals were included in the IBSC multicentre study; *n* = 13 at the psychiatric outpatient clinic and *n* = 17 at the Habilitations service centre. Of these, two from the psychiatric clinic and four from the Habilitation service centre did not complete their participation in the IBSC.

Coaches were all experienced in working with ASD and ADHD but came from different occupational backgrounds. At the psychiatric outpatient clinic coaches comprised two educational therapists, one clinical psychologist and one trained social worker. At the Habilitation service centre two special education teachers and one trained social worker were coaches. All of them were employed at the study centres.

### Participants and recruitment process for the interviews

A consecutive sampling method was applied. All individuals who took part in the IBSC multicentre study, including individuals who had dropped out, were asked to participate in the interview-study during visits at their home clinic. They received verbal and written information about the study and study personnel, and if they agreed to participate an interview date was set up at their home clinic. Depending on when the participants were enrolled in the original study, the interview could take place from three months and up to 21 months (median 8.5 months) after receiving the intervention. Seventeen participants agreed to be interviewed. One of these did not show up even though several interview dates were set up, resulting in a total of 16 individuals participating (See Table 1). Participants ranged in age from 15 to 32 years (mean = 23 [SD = 5]) and consisted of 56% males and 44% females. Reasons for declining participation were not shared. A monetary compensation for participation was offered, partly to cover any associated travel expenses.

**Table 1:** Clinical and sociodemographic characteristics of participants (*n* = 16)

Participant	Study Centre (1/2)	Diagnosis	Level of education (completed or ongoing)	Occupation	Civil state & living situation	Geographical Area	Completed participation in IBSC
1	1	ALC + ADHD	Compulsory School	Student	Single, living with parents	Rural	Yes
2	1	AS	Upper Secondary School	Sick leave	In a relationship, one person household	Urban	Yes
3	1	AA	Upper Secondary School	Student	Single, living with parents	Urban	Yes
4	1	ADHD	Upper Secondary School	Sick leave	In a relationship, co-habitation	Rural	Yes
5	1	AD + ADHD	Upper Secondary School	Student	Single, living with parents	Rural	Yes
6	1	ADHD + DCD	University	Student	Single, living with parents	Rural	Yes
7	2	AS	University	Student	In a relationship, one person household	Urban	Yes
8	2	AS + ADHD	Upper Secondary School	Student	Single, one person household	Urban	Yes
9	2	ALC + ADHD	Has not completed Compulsory School	Student	Single, living with parents	Urban	Yes
10	2	AS	Compulsory School	Student	Single, living with parents	Urban	Yes
11	2	AS + ADHD	Compulsory School	Work Experience Placement	In a relationship, one person household	Urban	Yes
12	2	ADHD	Upper Secondary School	Unemployed	Single, one person household	Rural	Yes
13	2	ADHD	Compulsory School	Working	Married, living with partner and/or children	Rural	No
14	2	AD	Upper Secondary School	Student	Single, living with parents	Urban	Yes
15	2	AS + ADHD	University	Work Experience Placement	Single, one person household	Urban	Yes
16	2	ALC	University	Unemployed	Single, one person household	Urban	Yes

*ADHD* attention-deficit/hyperactivity disorder, *ALC* autistic-like condition (pervasive developmental disorder not otherwise specified; PDD NOS), *AS* Asperger’s disorder, *AD* autistic disorder, *AA* atypical autism, *DCD* Developmental Coordination Disorder

### Data collection

Data was collected between the autumn of 2012 and spring of 2015 through single individual qualitative narrative interviews carried out at the clinic closest to their home. One interview took place in the home of the participant at their request. During one interview, the participant’s mother was present. A semi-structured interview guide was used. It included questions about thoughts; experiences and expectations participants may have had before, during and after receiving IBSC, benefits and disadvantages compared to other forms of support as well as likes and dislikes. The perceived significance of the coaches’ training and knowledge about ASD and ADHD was inquired about. The interviewer encouraged participants to speak freely and to regard the interview as a conversation with the purpose of capturing their subjective experience. Six interviews were conducted by the first author (HS), a female clinical psychologist employed at one of the study centres. As she had previously been involved as a coach and in the recruitment process at one of the study centres, she did not perform interviews with participants from her own clinic. Ten interviews were conducted by the second author (BHA), a senior female researcher with no previous contact or connection with any of the participants or involved clinics. The interviews had a median duration of 22 min. All interviews were anonymized, recorded and transcribed verbatim by professionals. Transcriptions were proof listened and read by the original interviewer to ensure the accuracy of the transcripts.

### Data analysis

Qualitative content analysis was used to analyze the interview data. This method lends itself well to the analysis and interpretation of the meaning in text and uses a systematic way of uncovering underlying patterns and themes [45]. It is also a flexible, inductive method, well suited for exploring participants’ perspectives regarding a specific research topic [46, 47]. The process involves dividing the text into meaning units that will then be condensed and abstracted into codes that label and capture the content. Codes are compared and interpreted into themes and subthemes. During the entire process, the text as a whole is to be considered [45, 47, 48].

The analysis started with the first author reading all transcribed interviews several times to provide a sense of the whole data. Then a stepwise process of dividing text into meaning units, codes and larger themes and sub-themes started. The qualitative software program NVivo v.10 was used as a complement to organize data. The analysis was an iterative process of cross-checking and testing categories against new data, revising them if necessary, while re-reading the original interviews ensuring that the initial meaning of a statement was preserved. Citations were chosen that were considered representative of themes and subthemes as well as including a range of participants. The first and second author continuously discussed the analysis process and the relevance of codes and the emerging themes, as well as together revising the analysis. In the case of discrepancy in interpretation, codes and themes were negotiated until agreement was reached. Finally, the codes under the different themes were counted in order to see if any specific diagnostic category completely dominated a certain theme or code. No such tendency was encountered. Examples of how the themes, sub-themes and codes were derived from the meaning units are shown in Table 2.

**Table 2:** Exemplification of the analysis and how the themes, sub-themes and codes were derived from meaning units

*Theme*	*Sub-theme*	Code	*Meaning unit*
Deciding to participate	Reflecting personal motives	Curiosity	*Interviewer: What did you think about the opportunity to receive internet-based support and coaching when you decided to participate in the project? From the start, when you decided to join… What did you think about it then, do you remember?* Participant 7: Wow. I thought it was really exciting to be able to participate in something like this, and… and I thought it sounded really exciting, to be able to try something new, an alternative to going to a clinic like you usually do *pauses*… that I thought was really exciting.
Taking part in the coaching process	Perceived short-term and long-term consequences	Improved self-confidence	*Interviewer: Is there anything … is there an area where you think* *“this works very well in my daily life”* *Participant 1: Yes, there is. Since I took part in this chat-project…* *Interviewer: Mmm…* *Participant 1: … I have become much more self-confident. I know that’s its okay to kind of withdraw when you feel that it’s all too much.*
The significance of format	Communicating through the written word	Missing personal interaction	*Participant 3: I think I felt the same about it then, as I feel about it now really. I felt that for me, for myself, I prefer it more personal. To sort of meet up and talk to each other.* *Interviewer: Mm* *Participant 3: I think you get more out of that.*

## Ethics approval and consent to participate

The study was approved by the Regional Ethical Review Board at the University of Gothenburg, Sweden (Dnr: 013–08; T364–10; T645–11; T436–12). For ethical considerations the guidelines of the World Medical Association Declaration of Helsinki (2008) were applied. Participants were given written and verbal information about the study. Written informed consent was obtained from all participants before the interview. All participants were deemed as ethically capable and of a level of maturity required for providing consent for themselves. All participants could easily and at any time during the interview receive support or medical supervision from their home clinic. None of the participants needed this.

## Results

Findings yielded three themes with a total of ten subthemes of relevance for the aims of this study (see Table 3). The themes were 1) Deciding to participate, with the subthemes *Reflecting personal motives* and *Defining necessary requirements for support;* 2) Taking part in the coaching process, with the subthemes *Defining coaching, Talking it through and receiving advice, Perceived short-term and long-term consequences;* 3) The significance of format, with the subthemes *Making support simple and accessible, Communicating through the written word*, *Being in one’s own home, A feeling of immediacy* and *Shortcomings and suggestions*.

**Table 3:** Themes and subthemes

Themes	Subthemes
Deciding to participate	*Reflecting personal motives* *Defining necessary requirements for support*
Taking part in the coaching process	*Defining coaching* *Talking it through and receiving advice* *Perceived short-term and long-term consequences*
The significance of format	*Making support simple and accessible* *Communicating through the written word* *Being in one’s own home* *A feeling of immediacy* *Shortcomings and suggestions*

### Deciding to participate

This theme describes the participants´ individual reasons for joining the study as well as reflections before participating.

#### Reflecting personal motives

Participants mentioned curiosity about a novel model as a main motive for participating. The model was seen as positive and progressive, the chat-format appealing to them as technologically familiar from their everyday life. It was also sometimes expected to be better suited to their situation;

*I thought being able to get in touch through the internet seemed particularly helpful as I experience face-to-face interactions as quite hard due to my Asperger-diagnosis. (Participant 1)*

Wanting to contribute to science and to the development of new “treatment” options that could benefit others were also mentioned, even when the intervention was judged as less relevant on a personal level. Furthermore, a need for support or help with issues specifically pertaining to one’s diagnosis or daily life problems, as well as simply wanting someone to talk to, were revealed as motives. The latter was often previously unfulfilled by for instance health care or family. Not having to travel to seek help was mentioned as a key motive.

#### Defining necessary requirements for support

Participants mentioned several requirements that were important in order to feel secure in the interaction with coaches and make participation feel meaningful. The necessity of receiving adequate information on the structure of the intervention and the chat-program was brought up, as was secure data transmission. A visual image of the person on the receiving end, along with meeting and getting acquainted with the coach were important so as to be able to share innermost thoughts and feelings. The principle of confidentiality between coaches and participants was also essential.

The coaches’ competence and knowledge were considered crucial. Formal training in health care and specific knowledge and experience of working with people with ASD and/or ADHD were viewed as ensuring understanding and trustworthiness, and was essential for asking questions concerning their disability. Participants hoped that the high competence level would avoid a repetition of previously negative experiences due to lack of understanding from others.

*I believe that people who don’t have knowledge about neuropsychiatry, don’t have as much of an understanding about the kind of problems you can experience, and therefore can’t really respond in a way that you need, especially if you’re going to receive coaching… I think most people who don’t have that kind of knowledge would be quite useless as coaches for people with neuropsychiatric problems, actually. (Participant 8).*

There were however exceptions to this where a committed, genuinely interested coach was viewed as more important than a high level of training, if this enabled wider availability of support in the future.

### Taking part in the coaching process

This theme reflects the participant’s thoughts on what they actually received in terms of support and coaching, what questions they discussed with their coach and reflections on which effects and consequences they experienced as a result of the process.

#### Defining coaching

“Coaching” was perceived as guidance in handling everyday issues. The coach was seen as an advisor who would help the participant with practical problem-solving, forming strategies, and reaching a better understanding of social interactions and how the outside world functioned.

*It means that somebody is with you all the way, showing you how to do something, telling you “this is how to do it” - whatever it is about (...) life, football or even reading the newspaper. It’s like somebody taking you by the hand and guiding you. (Participant 9).*

Although terms like “treatment” or “rehabilitation” were sometimes mentioned, there was a reluctance among participants to use such terms, emphasizing instead the more easy-going and supportive character of coaching, seeing the coach more as a helpful social contact.

*I don’t consider it a treatment, it’s more like someone helping you in general in your daily life. Like a friend that you don’t have a personal connection to. (Participant 6).*

#### Talking it through and receiving advice

Despite selecting subjects in advance, participants felt they could choose the chat-topic freely, sometimes preparing themselves by memorizing or writing down occurrences during the week. Participants mentioned talking through previous life experiences as well as more recent events. Help and advice were sought and received in the areas of planning and organization, problem-solving, and interpretation and management of social situations or conflicts. Some also discussed practical subjects such as medication. Topics were often related, directly or indirectly, to the participant’s diagnosis. The use of the support for processing feelings of insecurity and worry associated with a recent diagnosis of ASD or ADHD was mentioned.

*It came to be a lot about how I function in my everyday life. I need to piece myself together with what the diagnosis means and still keep track of who I am in the middle of everything. (Participant 4).*

#### Perceived short-term and long-term consequences

In the short term, always having someone “there” to turn to with thoughts and questions was reassuring. This was particularly valuable where it did not exist in one’s own network or where the individual was hesitant to seek support elsewhere, for example from healthcare.

*Fairly often it felt like you had something you wanted to ask about, that you didn’t really want to call someone and ask about. (….) I don’t call anyone, ever. (…) I really don’t like to talk at all. (Participant 15).*

Furthermore, chatting with someone had a calming effect on emotions;

*You could vent your anger, she listened to me. These things that (others) didn’t want to listen to, I got to talk about them there. And then, I must say, it helped to keep a more even mood. (Participant 12).*

The set-up of the intervention was described as lending itself to other areas of life, providing structure and valuable everyday reminders.

Long term consequences included improved planning skills and self-confidence, a sense of empowerment both in general and in social situations, and a better balance in life that is to say less stress and fatigue. Taking part in the project could also lead to learning to use writing as a general way of processing feelings and experiences.

Participants expressed being relatively satisfied with the intervention as a whole after the 8 weeks of the study, including length and frequency of chat sessions. Participants brought up having appreciated being offered IBSC in the future, that continuous support probably would have resulted in a greater effect. Difficulties in implementing advice, and doubting that the intervention had any lasting effects were also mentioned.

### The significance of format

The theme refers to how the participants perceived that the format and specific characteristics of the intervention impacted on their experiences of taking part in the study, as well as suggestions for improvements.

#### Making support simple and accessible

The flexibility and accessibility of the support were generally experienced as very positive. The simplicity of the program was appreciated and using a chat-program was a familiar form of communication. Time and effort were minimized compared for instance to taking time off work and travelling to a clinic. This was considered less troublesome, and enabled more frequent meetings.

*You don’t have to schedule a meeting, or travel to (the clinic) all the time - that type of thing. Given the fact that I have ADD, even getting here is quite a strain. It was simply a relief, being able to just sit down and start talking*. (*Participant 9*).

The same aspects were sometimes described as negative, leading to less commitment than to a physical meeting and even to forgetting about sessions. Views were also voiced that it was unimportant which form the support took, and that the special internet-based features could just as well have been replaced entirely by meetings or telephone calls.

#### Communicating through the written word

Communication through the written word was perceived as more accurate and precise, reducing the common concern of misunderstandings. Having the time to think things through and gather one’s thoughts stood out as particularly important.

*You can take your time and think about how to express yourself, and not be as worried about being misunderstood all the time. It made me feel considerably safer. (Participant 15).*

In contrast to the spoken word which was seen as fleeting and imprecise, what was written remained available on the screen for both parties, which also eliminated memory issues. Using the text-based format both in the preparation process (writing down questions or experiences) and in chat-sessions was perceived as simplifying. It was mentioned that it was sometimes easier to express oneself and to write down sensitive things rather than voicing them. However, a dislike of the text-based form of communication was also expressed and a feeling that an important part of the personal non-verbal interaction was lost.

#### Being in your own home

Being able to communicate in the privacy of one’s own home was seen as an advantage, alleviating stress and anxiety resulting from face-to-face meetings, social pressure and/or unfamiliar surroundings. This promoted better focus on the desired subject of discussion.

Another effect of not having to leave home was enabling communication even when feeling particularly tired or sad, or having a hard time going anywhere, thus reducing the risk of missed sessions or cancelled appointments.

*If I have a bad day I cancel the appointment, and it just doesn’t happen. But now I didn’t want to cancel, for me it wasn’t that difficult to use the chat-program. (Participant 4).*

However, chat-sessions were seen as a complement to, not a replacement for face-to-face interaction. A common view was that having to meet at a clinic was also innately worthwhile, even when one would rather stay at home.

#### A feeling of immediacy

Participants expressed a positive feeling of immediacy in relation to the intervention. This seemed to be an effect both of the higher frequency (two sessions a week) and of the e-mail function as a complement to chat-sessions. Participants experienced that they could reach their coach at almost any time without going through intermediaries e.g. a telephone operator. Communicating feelings immediately as for example in an e-mail was perceived as a great advantage despite the response being delayed, making it feel worthwhile and easier to stay on topic, and reducing the risk of forgetting. Furthermore, the higher frequency sometimes facilitated rapid implementation of given advice into daily life, knowing someone would soon follow up on what had been discussed.

*You were able to convey your thoughts and reflections in an email, in the exact same moment as you were thinking it. It’s not that easy to remember when you’re an “ADHD-kid”, you forget all the time (Participant 13).*

However, participants also expressed a wish for a more individually tailored chat schedule, varying the weekly frequency or cancelling scheduled sessions if they had nothing to discuss, thus avoiding frustrating “small-talk”.

#### Shortcomings and suggestions

Failing technology was a problem. Server malfunction without notification was confusing for the participants and led to some chat-sessions being cancelled, shorter than planned or carried out with difficulty. Such problems were often handled by means of a phone call from the coach and scheduling of a new session. Many, although frustrated, were tolerant about this as they knew that the program was under development.

Participants suggested automatically updated information about server-status. Furthermore, access to chat-logs between sessions would make it easier to remember and process what had been discussed. A smart-phone application was suggested in order to enable use in any situation, further increasing accessibility. An “on call”-chat service would also have been appreciated.

Participants felt that engaging in activities outside of one’s home was important and would presumably result in better effect, offering situations for challenging themselves or practicing skills.

*If you had a few more daily activities (..), I think it would be an even better help. Because then you get a little more exposure to things you maybe don’t like and so on, (…) and then I think it could result in an even better effect (….) given you simply have more to process (Participant 10).*

There was a wish to be able to vary the form of communication between live meetings, chat sessions, email-conversations, video conversations or even group-chat sessions, the latter being helpful when more than one profession was needed or as a way of gathering people with similar problems for discussions. Also mentioned as having been appreciated was help in informing other concerned parties about the program (for example an employer) so as to minimize disruptions.

## Discussion

Using a qualitative methodology, this study aimed to gather in-depth information about how adolescents and adults with ADHD and/or ASD experienced an 8-week internet-based support and coaching model. It also aimed to consider how such a format could benefit individuals that often struggle with traditional and existing support options. A number of interesting findings emerged, showing how specific qualities of the intervention corresponded to the needs of this particular group of patients, both in terms of the internet-based format as well as their experienced need for support in general. Findings also highlighted important requirements for receiving support and suggestions for improvements.

The first theme, “*Deciding to participate*”, showed reasons for participating including expecting the internet-based model to be better suited to their situation in the light of an ASD and/or ADHD diagnosis. However, motives could also be related to previously unmet needs for support or wanting to discuss issues pertaining to living with such a disorder. Prior studies suggest that an experience of insufficient support might be common among individuals with both ASD and ADHD, especially when it comes to non-medical alternatives [16, 17, 49, 50]. Participants further felt that coaches’ knowledge and experience of these disorders were pivotal and a necessary requirement in deciding to participate. This stemmed largely from prior negative experiences both in health care and in daily life. Such negative experiences have been recognized previously in studies concerning individuals with both ASD and ADHD, often resulting in perceived stigma and a strong desire for more knowledge and raised awareness within these contexts [16, 51–53]. College students with ADHD have described experiencing a lack of knowledge about their disorder among service providers and faculty, these also being perceived as ill equipped to meet their needs [18]. This further stresses the importance of specific knowledge and experience among professionals delivering this kind of support [18].

Findings relating to the theme, “*Taking part in the coaching process*”, showed that participants defined coaching as easy going, as a social contact and as guidance to everyday life concerns. They were also reluctant to the use of terms such as “treatment”. In a study by Lefler, Saccetti and Del Carlo [18] students with ADHD showed a preference towards receiving help in the form of coaching as compared to when the term “therapy” was used. This could mean that IBSC might be a more appealing form of support for this kind of individual. Our participants also largely utilized the support to discuss issues directly or indirectly related to their diagnosis. Defining coaching as guidance on issues relating to ASD and/or ADHD corresponds well with the original intentions when developing the IBSC model [12]. Viewing it as a social contact could imply that it was unclear for participants what differentiated the professional support from more informal help. The expressed importance of education and knowledge among coaches however partly contradicts this. Findings further made it apparent that participants appreciated and experienced a need for this kind of support after recently undergoing a neuropsychiatric evaluation as this could raise feelings of worry or uncertainty. A diagnosis of ADHD in adulthood can have great emotional impact, raising concerns about one’s identity [54, 55]. Psychoeducation, with the development of compensational strategies, is an important intervention in these cases [56] as in several other psychiatric disorders [57]. Receiving psychoeducation has also been shown to be a strong predictor for overall satisfaction with treatment in ADHD [58]. The intention with IBSC was to offer personalized support and coaching, as well as individualized psychoeducation in order to aid participants in creating a better understanding of their functioning connected to a diagnosis of ADHD and/or ASD. IBSC might thus be a helpful option after newly receiving a diagnosis of ASD or ADHD.

There was an expressed threshold among participants for seeking help from health care or in their own social network. This is in line with a qualitative study in which young adults with ADHD hesitated to seek and receive help as an effect of perceived stigma [18]. Problems for individuals with ASD in initiating social interactions could also contribute to this [59] as well as more general problems with initiative in ADHD [60]. Hence, a valued effect of the intervention was the reassurance of having “someone to turn to” with thoughts and questions. In relation to this there was a tendency to report mostly short-term effects, such as emotional comfort and “on-tap” reassurance. Behaviors resulting in short term relief of anxiety or worry can often maintain such problems in the long run and are therefore not always advisable [61]. On the other hand, the aim of IBSC was primarily information and help with basic strategies related to living with ASD and ADHD, targeting a lack of adaptive skills more prominent in these populations. The intervention can also be considered valuable as an option for reaching these individuals at all, considering the various and shared difficulties and obstacles to seeking and receiving help. Some long term positive consequences as a result of the intervention were mentioned, namely improved planning proficiency, self-confidence and less stress and fatigue. These were in line with results from the pilot study [12].

The theme “*The significance of format*” revealed that participants viewed communication through the written word as largely helpful, offering time to think things through, promoting precision and reducing the risk of misunderstandings. Two previous qualitative studies have highlighted the aspect of writing as a helpful way of communicating for adults with ASD [10, 59]. The ASD-characteristic of slow processing speed has also been shown to present problems in face-to-face communication for these individuals [10], suggesting that written communication could be useful for them. Memory issues during an ongoing chat-session were likewise alleviated, an important advantage considering the fact that working memory-deficits are a problem for individuals with ADHD [62]. Written communication was moreover seen as helpful for disclosing sensitive issues, as shown in previous studies of internet -based treatment [63].

Being in the privacy in one’s own home was experienced by our participants as reducing social pressure and anxiety, and promoting better focus on the subject at hand. Although not specifically mentioned, it is not unlikely that sensory issues can in part have contributed to this result. Nicolaidis et al. [10] found that sensory sensitivities such as being disturbed by the lighting or having difficulties filtering out background noises were a prominent problem for adults with ASD within a traditional health care situation. This can be alleviated by home based IBSC. In our study, being able to communicate from one’s home was further found to lead to fewer appointment cancellations due to for example having “a bad day”. In adolescents and adults with ADHD, difficulties with initiative, planning and memory can affect treatment adherence and high dropout rates are common [35, 36]. Individuals with ASD have furthermore been shown to experience unpredictable and variable mood swings which sometimes present a complete barrier to their activities [52, 64]. In such circumstances, having the possibility to communicate from home is probably an advantage. However, whilst appreciating the home aspect, participants saw meeting face-to-face as valuable and health promoting and online support as a complement to, not a replacement for this. This is in line with results in the pilot study [12]. Responses also indicated that participants felt that some kind of parallel activity outside of the program was important in order to practice skills or challenge themselves to boost the effect. Practicing skills and exercises promoting behavior change are recognized as important components in for example cognitive behavior therapy [61] and social skills training for autism [65] and could thus be important to consider.

The simplicity and accessibility of the support, along with perceived immediacy in contact with the coach, were appreciated features and have been recognized as hallmarks of internet-based solutions [63]. In individuals with ADHD, immediacy and ease in receiving support might generally be key factors in promoting adherence. Also valued was the higher contact frequency facilitating rapid implementation of advice and reducing the risk of forgetting. However, it was also mentioned how the simplicity of the support could in some instances lead to less commitment and not taking the intervention as seriously as would a clinic appointment, which is also consistent with previous knowledge [63].

Disadvantages with the IBSC included experiencing incomplete personal interaction. Also, failing technology was a problem and is important to manage before using the model on a wider scale. Difficulties in implementing advice were voiced, although for some participants the high session frequency seemed to facilitate this. It is unclear if problems of this kind are specific to this support format. Finally, suggestions from participants in the present study included a greater flexibility in support frequency, chat-support “on demand”, and being able to vary the form of communication between for example live-meetings, chat, email, video, and group-chat. A desire for practical support on hand has been voiced in previous qualitative articles, where participants suggested that a helpline would be useful to handle unexpected situations and difficult emotions [10, 52]. However, this was tried out in part when developing the original IBSC model with less success [12].

### Trustworthiness and limitations

To ensure trustworthiness in this qualitative study certain aspects were important to consider, especially the concepts of credibility and transferability [46]. Credibility refers to how well the method and research process is suited to and answers the research question, making the results believable [66, 67]. To attain credibility in this study we strove to provide a detailed, clear and transparent description of the whole research process, including the gathering of data, the process of analysis and subsequent results. To clarify relations between results and the original data, representative citations were chosen. There was a consistent dialogue between co-researchers during the whole process. Transferability describes to which degree the results can be transferred to other settings [46, 66, 67]. In this study detailed information on the context, participants and interview questions were disclosed to ensure transferability.

In relation to our results the following limitations need to be considered. Firstly, the analysis was carried out including all diagnostic categories (ADHD, ASD or ADHD and ASD in combination). Hence, it does not lend itself well to distinguishing issues or responses that differentiate the ADHD/ASD groups and how specific characteristics of IBSC suited one or the other diagnostic category. However, we also know that there is a considerable overlap between the disorders, that there are often sub-clinical ASD or ADHD-traits present and that there are shared cognitive difficulties in several domains between disorders [7, 68–70]. This questions the value of a separate analysis procedure. Although the current study does not have a quantitative approach, when results were final a count was performed to see if any specific diagnostic group completely dominated a certain code or theme. No such tendency was found.

Another limitation is that for some of the participants, a significant amount of time had elapsed between receiving the intervention and taking part in the interview. This was due to the intervention taking place between the autumn of 2010 and winter 2014, and the decision to carry out an additional qualitative interview study being made in 2012. This could affect how well some participants remembered certain aspects. However, research has shown that adults with ADHD can in fact accurately recall childhood ADHD symptoms, which could imply that recollection might not be that affected [71]. Also, the aim of this study was not to render accurate or true remembrance but rather the meaning and significance of the participants’ experiences.

The first author had previously been involved as a coach and in recruiting participants at one of the study centres. No interviews were for this reason conducted by the first author at this site. There remains a possibility that preconceptions could affect the analysis phase. On the other hand, in qualitative research this does not necessarily equal bias as long as the researcher attends to it and fosters a process of reflexivity, i.e. regarding the effect of the researcher and how knowledge is constructed during the different phases of the research process [72]. It can then contribute to a likewise valid understanding of the subject of research. To promote reflexivity the data was also cross-examined by the second author during the whole process.

Not all participants wanted to go through with the interview unfortunately, but all of them did get the opportunity; hence it was not a convenience sample. This is, however, the first time that the participants themselves get to voice their opinions about the intervention (IBSC) described in the pilot study [12].

## Conclusions

This qualitative study provides in-depth data about the experiences of adolescents and young adults with ADHD and/or ASD taking part in an internet-based support and coaching model. The findings highlight an appreciation for the IBSC model and demonstrate that it was in many ways a helpful method of communication for these individuals, as well as offering them support in handling daily life problems. It was not however seen as a substitute for face-to face interaction. The findings imply that IBSC has specific advantages for adolescents and young adults with ASD and/or ADHD. It could therefore be an important complement to standard treatment and support options, especially regarding increased adherence to offered support and as a way of reaching those who experience thresholds for seeking and receiving help.

## Abbreviations

ADD: Attention Deficit Disorder
ADHD: Attention deficit hyperactivity disorder
ASD: autism spectrum disorder
DSM: Diagnostic and Statistical Manual of Mental Disorders
ESSENCE: Early Symptomatic Syndromes Eliciting Neurodevelopmental Clinical Examinations
IBSC: internet-based support and coaching
